# Особенности иммунологического статуса пациенток с аменореей (обзор литературы)

**DOI:** 10.14341/probl13456

**Published:** 2024-06-04

**Authors:** Ю. С. Абсатарова, Ю. С. Евсеева, Е. Н. Андреева, З. Т. Зураева, Е. В. Шереметьева, О. Р. Григорян, Р. К. Михеев

**Affiliations:** Национальный медицинский исследовательский центр эндокринологии; Национальный медицинский исследовательский центр эндокринологии; Национальный медицинский исследовательский центр эндокринологии; Российский университет медицины; Национальный медицинский исследовательский центр эндокринологии; Национальный медицинский исследовательский центр эндокринологии; Национальный медицинский исследовательский центр эндокринологии; Национальный медицинский исследовательский центр эндокринологии

**Keywords:** аменорея, иммунная система, синдром поликистозных яичников, преждевременная недостаточность яичников

## Abstract

Аменорея — распространенный симптом целого спектра нозологий среди женщин репродуктивного возраста, который может сопровождать любую эндокринопатию в стадии декомпенсации. Во всем многообразии различных звеньев патогенеза нарушений репродуктивной функции проблема иммунопатологии остается немного в стороне, однако значимость этих расстройств недооценена. Настоящая публикация представляет собой обзор нарушений в иммунной системе у женщин с аменореей.Как известно, при синдроме поликистозных яичников (СПЯ) и преждевременной недостаточности яичников (ПНЯ) одним из клинических проявлений является аменорея. С одной стороны, эти нозологии существенно отличаются друг от друга по этиологии, патогенезу и подходам к терапии, а с другой — у них есть сходство, проявляющееся иммунологическими нарушениями. В статье приведены сведения об иммунном статусе пациенток с СПЯ и ПНЯ. Проанализированы работы, посвященные различным расстройствам в иммунной системе, патологиям гуморального и клеточного иммунитета, которые в перспективе могут послужить ключом к разработке новейших и нестандартных методов лечения таких социально значимых заболеваний.Поиск литературы проводили в отечественных (eLibrary, CyberLeninka.ru) и международных (PubMed, Cochrane Library) базах данных на русском и английском языках. Выбор источников был приоритетен периодом с 2018-го по 2024 гг.

## Abstract (en)

Amenorrhea is a common symptom of a whole range of nosologies among women of reproductive age, which can accompany any endocrinopathy in the stage of decompensation. In all the diversity of various links in the pathogenesis of reproductive disorders, the problem of immunopathology remains a little aside, however, the significance of these disorders is underestimated. This publication provides an overview of immune system abnormalities in a women with amenorrhea. As is known, in polycystic ovary syndrome (PCOS) and premature ovarian insufficiency (POI), one of the clinical manifestations is amenorrhea. On the one hand, these nosologies differ significantly from each other in etiology, pathogenesis and approaches to therapy, and on the other hand, they have a common similarity, manifested by immunological disorders. The article provides information about the immune status of patients with PCOS and POI. Works devoted to various disorders in the immune system, pathologies of humoral and cellular immunity, which in the future may serve as the key to the development of new and non-standard methods of treating such socially significant diseases, are analyzed. Literature search was carried out in national (eLibrary, CyberLeninka.ru) and international (PubMed, Cochrane Library) databases in Russian and English. The choice of sources was prioritized for the period from 2018 to 2024.

## ГЛОССАРИЙ

Лимфоциты — ключевые клетки адаптивного иммунного ответа, обеспечивающие гуморальный и клеточный иммунитет и регулирующие деятельность других иммуноцитов, участвующих в защите организма. Различают следующие виды клеток:

1. Т-лимфоциты — тип лимфоцитов, участвующих в основном в реакциях клеточного иммунитета.

• Т-хелперные (Th) клетки (CD4+) стимулируют пролиферацию и дифференцировку как Т-, так и В-лимфоцитов, секретируя цитокины. В зависимости от того, какие цитокины они продуцируют, среди них различают:

· Th1 (Т-хелперы первого типа) — секретируют интерлейкин-2 (IL-2), IL-6, IL-8, интерферон-гамма (IFN-γ), фактор некроза опухоли-альфа (TNF-α), обеспечивают реакции Т-клеточного иммунитета: стимулируют иммунный ответ против внутриклеточных бактерий, противовирусный, противоопухолевый, трансплантационный иммунитет;

· Th2 (Т-хелперы второго типа) — секретируют IL-4, IL-5, IL-6, IL-10, IL-13 и активируют синтез антител, способствуют развитию гуморального иммунного ответа против внеклеточных бактерий и их токсинов;

· Th17 клетки выполняют провоспалительные функции, высвобождая уникальный спектр цитокинов (IL-17, IL-21, IL-22, IL-26 и TNF-α), и обеспечивают защитные реакции организма.

• Цитотоксические (Tc) Т-клетки (CD8+) способны специфически осуществлять лизис клеток-мишеней. Их роль велика в реализации трансплантационного иммунитета, развитии аутоиммунных заболеваний и в противоопухолевой защите.

• Регуляторные (Treg) клетки выполняют важную функцию завершения адаптивного иммунного ответа и обеспечения толерантности к собственным антигенам. В сущности, эти клетки являются супрессорами и могут подавлять активацию, пролиферацию и эффекторные функции широкого круга иммунокомпетентных клеток.

2. B-лимфоциты — тип лимфоцитов, участвующих в реакциях гуморального иммунитета.

3. Клетки естественные киллеры (NK) — группа лимфоцитов врожденного иммунитета, обладающих цитотоксической активностью в отношении опухолевых клеток без предварительной сенсибилизации и вызывающих гибель инфицированных вирусами клеток. Секретируют различные цитокины и регулируют активность других иммунных клеток.

## ВВЕДЕНИЕ

Нарушения менструального цикла являются одной из самых частых причин обращения женщин к акушеру-гинекологу [1]. Аменорея — это состояние, представляющее собой отсутствие менструации. В зависимости от наличия менархе выделяют первичную и вторичную формы: при первичной менархе отсутствует. Вторичная аменорея определяется как отсутствие менструации более 3 месяцев при регулярном менструальном цикле или более 6 месяцев при нерегулярном. По оценкам Всемирной организации здравоохранения, нарушения менструальной функции, и в частности аменорея, являются шестой по значимости причиной женского бесплодия. Среди женщин репродуктивного возраста распространенность вышеописанного симптома варьирует от 5 до 13% [2].

Иммунологические нарушения являются неотъемлемой частью гинекологических заболеваний, сопровождающихся отсутствием менструаций, особенно это касается таких нозологий, как синдром поликистозных яичников (СПЯ) и преждевременная недостаточность яичников (ПНЯ). В последние десятилетия вопрос о роли иммунопатологии в работе репродуктивной системы активно изучается. Понимание сложнейших процессов регуляции клеточного и гуморального иммунитета в рамках гинекологической эндокринологии позволяет не только формулировать новые теории патогенеза заболеваний, но способствовать созданию возможных инновационных схем терапии.

Целью работы является предоставление обзора литературы по изучению иммунного статуса пациенток с аменореей и, как следствие, о возможном применении полученных теоретических знаний по иммунопатологии на практике в лечебных целях.

## ВЛИЯНИЕ ПОЛОВЫХ ГОРМОНОВ НА ИММУННУЮ СИСТЕМУ

Прежде чем приступить к описанию особенностей работы иммунной системы при репродуктивных заболеваниях, уделим внимание непосредственному влиянию половых гормонов на иммунитет. Клеточными эффекторами иммунной системы являются лейкоциты, к которым относятся лимфоциты, моноциты, макрофаги, дендритные клетки (ДК), нейтрофилы, базофилы и эозинофилы. Лимфоциты можно разделить на подклассы на основании функций и по маркерам клеточной поверхности; эти маркеры называют кластерами дифференцировки (CD). Подклассы лимфоцитов включают Т-клетки, В-клетки и NK-клетки [3].

Т-лимфоциты выполняют различные биологические функции, в основном участвуют в клеточном иммунном ответе организма. Эти клетки модулируют врожденные иммунные реакции. Кроме того, они могут влиять на все иммуноциты, такие как ДК, гранулоциты, NK клетки и миелоидные супрессорные клетки. Т-лимфоциты можно разделить на три подмножества, такие как Th-клетки, Tc-клетки и Тreg-клетки [4].

Андрогены оказывают противовоспалительное действие и могут подавлять активность иммунных клеток. Супрессивная роль мужских половых гормонов проявляется подавлением функционирования ДК и экспрессией их костимулирующих маркеров в лимфатических узлах, снижением секреции IFN-γ, провоспалительных цитокинов макрофагами и ослаблением ответа Тh1-лимфоцитов. Андрогены вызывают значительное снижение экспрессии toll-подобного рецептора 4 (TLR4) на поверхности макрофагоподобных клеток. Более того, тестостерон подавляет дифференцировку В-клеток и выработку антител [5]. Один из механизмов, посредством которого андрогены проявляют свое противовоспалительное действие, является активация протеин-тирозинфосфатазы 1 (Ptpn1), в результате чего образуется комплекс с тестостероном, который дефосфорилирует рецепторы к IL-12, ингибируя тем самым его сигнал, имеющий решающее значение для дифференцировки Th1-лимфоцитов [6]. Кроме того, мужские половые гормоны могут регулировать адаптивный иммунитет у людей путем подавления активности Th2 и Th17-клеток, но индуцируя функционирование Treg-клеток [7]. Однако противовоспалительные эффекты андрогенов не являются повсеместными. Гиперандрогения при СПЯ, напротив, поддерживает воспаление путем регуляции количества макрофагов и их фенотипов. Более высокие соотношения воспалительных М1 макрофагов к противовоспалительным M2 макрофагам наблюдаются в яичниках экспериментальных моделей с данным синдромом [8].

Эстрогены оказывают более сложное регулирующее действие на иммунную систему. В отличие от андрогенов женские половые стероиды считаются мощными активаторами защитной функции организма. Поскольку уровни стероидных гормонов колеблются на протяжении всего менструального цикла, высокая концентрация эстрогенов может подавлять синтез провоспалительных цитокинов во время и после овуляции и потенцировать воспалительный процесс в период ранней фолликулярной фазы, когда уровень этих стероидов падает [9]. Эстрадиол оказывает стимулирующий эффект на выработку Т-клетками IFN-γ и TNF-α при его невысоких значениях, а также на IL-4 и IL-10, когда концентрация гормона повышается. В постменопаузе, когда имеется явный эстрогендефицит, последний способствует выработке IL-1 ДК, тогда как во время беременности гиперэстрогения ингибирует синтез IL-1, IL-6 и TNF-α [10]. Важность женских половых гормонов для развития и функциональности Т-клеток подчеркивается заметными различиями в распространенности и тяжести аутоиммунных заболеваний у мужчин и женщин. Интересно, что эстрогены оказывают бимодальный эффект на патологию аутоиммунных заболеваний, опосредованных Т-клетками (рассеянный склероз, атопический дерматит, первичный билиарный цирроз): высокий уровень этих гормонов (например, при беременности) ингибирует, а низкий — стимулирует функцию Т-лимфоцитов. Неудивительно, что эти патологии чаще встречаются у женщин в постменопаузе [11].

Почти при любой концентрации эстрогены активируют образование антител В-лимфоцитами, тем самым предрасполагая к развитию аутоиммунных заболеваний. Имеются данные о связи женских половых гормонов с индукцией аутоагрессивных В-клеток, что может объяснить, почему определенные аутоиммунные заболевания, опосредуемые В-лимфоцитами (системная красная волчанка, миастения), более распространены у женщин в репродуктивный период [12].

## СИНДРОМ ПОЛИКИСТОЗНЫХ ЯИЧНИКОВ

СПЯ является наиболее распространенным гинекологическим, эндокринным и метаболическим заболеванием у женщин репродуктивного возраста. Данная патология характеризуется овуляторной дисфункцией, гиперандрогенией и морфологической картиной множественных кист яичников, и в настоящее время является основной причиной ановуляторного бесплодия [13][14]. Все больше публикаций свидетельствует о том, что вялотекущее хроническое воспаление играет ключевую роль в возникновении и развитии этого гетерогенного заболевания [15].

Многочисленные исследования показали, что системное хроническое воспалительное состояние у женщин с СПЯ характеризуется повышением уровней цитокинов (IL-6, IL-18, TNF-α) и белков острой фазы (высокочувствительный С-реактивный белок [hs-СРБ], белок теплового шока 70) в периферической крови [16]. Считается, что вялотекущее хроническое воспаление у больных овариальной гиперандрогенией связано с висцеральным ожирением. Более 50% пациенток с данным синдромом имеют избыточный вес, в то время как ожирение индуцирует адипотоксичность, приводящую к выбросу большого количества цитокинов (С-реактивный белок (СРБ), TNF-α и IL-6) и адипокинов в кровь [17]. Следовательно, ожирение может влиять на воспалительный статус женщин с СПЯ. Существуют также маркеры эндотелиального повреждения, такие как асимметричный диметиларгинин (ADMA), гомоцистеин, ингибитор активатора плазминогена-I (PAI-I) и фактор роста эндотелия сосудов (VEGF) [18]. Эндотелиальная дисфункция и измененный цитокиновый профиль в сторону провоспалительных и протромботических состояний при СПЯ могут увеличивать сердечно-сосудистый риск и в значительной степени ограничивать репродуктивную способность (начиная от ановуляции и заканчивая плацентарной недостаточностью) [19]. Кроме того, лечению овуляторной дисфункции препятствует тот факт, что аномальные уровни TNF-α, ангиопоэтина-2 и адипонектина могут быть связаны с резистентностью к кломифена цитрату у больных [20].

Известно, что различный количественный и качественный состав цитокинов оказывает влияние на инициацию адаптивного иммунного ответа путем дифференцировки наивных CD4+ Т-лимфоцитов в Т-хелперы (Th1, Th2, Th17) и подмножества Treg-клеток путем активации Т-клеточного рецептора (TCR). Из-за большого количества фолликулов у женщин с СПЯ повышаются уровни эстрогенов, которые усиливают секрецию воспалительных цитокинов клетками Th1, таких как IL-6, TNF-α и IFN-γ. IL-6 способствует превращению Th0-клеток в Th17-клетки, которые также являются провоспалительными [21]. Имеются данные, указывающие на то, что в дополнение к увеличению количества провоспалительных Th17-клеток в кровотоке у женщин с СПЯ значительно снижается количество противовоспалительных Treg-клеток. Примечательно, что дисбаланс клеток Th17/ Treg является значимым фактором в патогенезе заболевания [22]. Дисбаланс про- и противовоспалительных Т-лимфоцитов и соответствующих им цитокинов усиливает воспалительный ответ, который синергически усугубляет хроническое воспаление.

В яичниках и жировой ткани наиболее распространенными иммунными клетками являются макрофаги. Они необходимы для поддержания баланса между деструктивным и защитным иммунитетом при воспалении. Уровни макрофагов варьируют на протяжении всего менструального цикла и являются самыми высокими во время овуляции и в лютеиновой фазе, таким образом, прогестерон оказывает на них непосредственное влияние. Многие женщины с СПЯ имеют избыточную массу тела или ожирение — патологические состояния, при которых макрофаги трансформируются из противовоспалительного фенотипа M2 в провоспалительный M1 [23]. Макрофаги M1 синтезируют воспалительные цитокины, такие как IL-1, IL-6 и TNF-α, присутствующие в высоких концентрациях в сыворотке и в фолликулярной жидкости больных. Овариальная гиперандрогения, вероятно, приводит к трансформации этих клеток в состояние M1, к повышению уровня цитокинов, что усугубляет инсулинорезистентность, нарушает регуляцию гипоталамо-гипофизарно-яичниковой оси и усиливает выработку мужских половых гормонов, замыкая порочный круг [24].

Примечательной является работа J. Shang и соавт., в которой изучались воспалительные процессы внутри яичников и их влияние на качество эмбрионов у пациенток с СПЯ и нормальным индексом массы тела (ИМТ), вступивших в протокол экстракорпорального оплодотворения (ЭКО) [25]. В ходе исследования авторы оценили 34 цитокина и зарегистрировали, что у больных значительно повышены уровни воспалительного белка-1β макрофагов (MIP-1β) и фактора-1α стромальных клеток (SDF-1α) в фолликулярной жидкости по сравнению с группой контроля. Стоит отметить, что MIP-1β представляет собой хемокин CC типа Th1, который вырабатывают в основном активированные моноциты [26]. В своей работе J. Shang и соавт. обнаружили повышенное количество моноцитов в периферической крови пациенток с СПЯ и нормальным ИМТ, что может быть связано с более высоким уровнем MIP-1β [25]. Последний по-разному воздействует на иммунные и неиммунные клетки при разных патофизиологических условиях. Имеются данные, указывающие на то, что MIP-1β может способствовать развитию опухолей путем индукции опухоль-ассоциированных макрофагов, Т-клеточной инфильтрации и секреции других хемокинов для подавления противоопухолевого иммунитета. И наоборот, он может усиливать данный вид иммунитета посредством привлечения макрофагов с фагоцитарной активностью и цитолитических лимфоцитов [27]. Кроме того, была показана обратная корреляция уровня MIP-1β в фолликулярной жидкости и количества эмбрионов хорошего качества D3, а также частоты образования бластоцист хорошего качества у пациенток с СПЯ (p=0,006 и p=0,003 соответственно). Ранее сообщалось, что концентрации MIP-1β взаимосвязаны с характеристиками яйцеклеток и исходами беременности [28]. Исследователи предположили, что уровень MIP-1β можно использовать как потенциальный биомаркер, связанный с качеством эмбрионов у женщин с СПЯ и нормальным ИМТ.

SDF-1α первоначально был идентифицирован как пре-В-клеточный фактор роста (PBGF) и считается наиболее мощным хемокином, поскольку может активировать миграцию и/или рекрутировать различные лейкоциты, включая лимфоциты, моноциты, гемопоэтические стволовые клетки и клетки-предшественники. Кроме того, in vitro было показано, что SDF-1α ингибирует апоптоз клеток в клеточной линии гранулезного рака яичников человека и играет важную роль в эмбриогенезе [29]. J. Shang и соавт. обнаружили, что уровни SDF-1α, как правило, отрицательно коррелировали с уровнем фолликулостимулирующего гормона (ФСГ), хотя корреляция была слабой. А уровни SDF-1α не имели связи с качеством эмбрионов у пациенток с СПЯ. Кроме того, концентрация данного хемокина была значительно ниже у пациенток с высоким уровнем андрогенов, чем в контрольной группе (p=0,031) [25]. Данный факт позволяет предположить, что SDF-1α тесно связан с гиперандрогенией и может играть важную роль в регуляции репродуктивной функции у больных СПЯ.

Группа ученых под руководством N. Aru провели рандомизацию по Менделю (MР) — это метод исследования, который использует генетические варианты в качестве инструментальных переменных, позволяющих изучить причинно-следственную связь между фактором риска и интересующим исследователя заболеванием. В данной работе использовали комплексный МР-анализ с двумя выборками для изучения причинно-следственной связи между 731 иммунной клеткой и СПЯ [30]. Результаты показали, что два типа В-клеток, а именно CD20-CD38- и В-клетки памяти, были достоверно связаны с повышенным риском развития овариальной гиперандрогении. В работе A. Ascani и соавт. была определена важная роль В-клеток в патогенезе СПЯ [31]. Известно, что В-лимфоциты продуцируют антитела при контакте с антигенами, и образование комплексов антиген-антитело может способствовать воспалительным реакциям в организме, тем самым потенциально увеличивая риск этого заболевания [31]. CD20 — это особый антиген, обнаруживаемый на поверхности В-лимфоцитов, и он известен своим участием в регуляции процессов пролиферации, дифференцировки и передачи сигналов [32]. Кроме того, в одной из работ была установлена корреляция между повышенными уровнями CD39+CD4+Treg-клетками и сниженным риском СПЯ. Регуляторные Т-клетки особенно важны для реализации репродуктивной функции. Их увеличение наблюдается во время нормальной беременности, а снижение повышает риски выкидыша или бесплодия у пациенток с СПЯ [33]. В данном исследовании авторы обнаружили корреляцию между уровнем CD14-CD16+-моноцитов и повышенным риском развития этого синдрома [30]. Моноциты и макрофаги действуют как «часовые» иммунной системы, и их можно отличить по экспрессии CD14 и CD16. Подмножества моноцитов классифицируются на основе фенотипических маркеров: классические (CD14+CD16-), промежуточные (CD14+CD16+) и неклассические (CD14−CD16+) [34]. Результаты исследования N. Aru и соавт. подтвердили участие неклассических моноцитов в манифестации СПЯ [30]. По сравнению с классическими моноцитами подгруппа CD16+ демонстрирует более сильную способность высвобождать провоспалительные факторы и, как следствие, повышена у пациенток с гиперандрогенией [30][35]. Патогенез СПЯ многогранен, и клиническая гетерогенность различных типов иммунных клеток, участвующих в возникновении и развитии синдрома, очевидна. Необходимы дальнейшие исследования для изучения взаимодействия между иммуноцитами и процессами врожденного и приобретенного иммунного ответа у пациенток с данной нозологией.

## ПРЕЖДЕВРЕМЕННАЯ НЕДОСТАТОЧНОСТЬ ЯИЧНИКОВ

Преждевременная недостаточность яичников (ПНЯ) — патология, сопровождающаяся истощением овариального резерва яичников в возрасте до 40 лет, аномальными менструациями (аменорея, редкие или частые менструации), повышенным уровнем ФСГ (>25 МЕ /л) и сниженным уровнем эстрадиола. Частота данной нозологии составляет 1–2% среди женщин репродуктивного возраста [36]. Этиопатогенез заболевания в большинстве своем остается невыясненным, однако в ряде случаев выявляют генетические аномалии, ятрогенные факторы, аутоиммунные заболевания, нарушения обмена веществ и инфекции [37].

Особенностью патологии является дисфункция гранулезных клеток, избыточный апоптоз которых может вызвать фолликулярную атрезию и привести к ПНЯ. Помимо ооцитов и клеток гранулезы, микроокружение фолликулов также составляют иммуноциты и цитокины. Взаимодействие между иммунными клетками, ооцитами и соматическими клетками опосредованы каскадом цитокинов и факторов роста, участвующих в процессе роста фолликулов, овуляции, лютеинизации, а также в стероидогенезе [38]. Многочисленные исследования выявили лимфоцитарную инфильтрацию и другие иммунные реакции у пациенток с ранним истощением овариального резерва. Воспаление в яичнике может ухудшать их функцию [39]. Тем не менее закономерности инфильтрации иммунными клетками все еще нуждаются в дальнейшем изучении.

Иммунная система имеет решающее значение для поддержания гомеостаза яичников и нормального функционирования репродуктивной системы. В одном исследовании был зарегистрирован повышенный уровень цитокинов, таких как макрофагальный воспалительный белок-1α (MIP-1α), IL-8 и индуцируемый интерфероном-γ белок-10 (IP-10), в фолликулярной жидкости пациенток с ПНЯ. Авторами был сделан вывод о том, что измененный цитокиновый профиль с повышенным содержанием хемокинов и факторов роста был связан с ранней стадией этого заболевания [40]. В другой работе было показано, что дефицит Treg-клеток и усиленный аутоиммунитет Th1-лимфоцитов могут участвовать в манифестации описываемой патологии. Цитокины, вырабатываемые Th1-клетками, IFN‐γ и TNF‐α, непосредственно стимулировали апоптоз и ингибировали пролиферацию и стероидогенез в гранулезных клетках человека in vitro, подавляя фактор роста соединительной ткани (CTGF) и цитохром P450 семейства 19 (CYP19A1) [41].

Макрофаги являются одной из преобладающих популяций иммунных клеток, участвующих в фолликулогенезе и овуляции. Провоспалительная микросреда, связанная с макрофагами M1, может влиять на жизнеспособность гранулезных клеток человека [42]. Стоит отметить работу D. Liu и соавт., в которой была выявлена высокая концентрация моноцитов и макрофагов M1 в фолликулярной жидкости у пациенток с ПНЯ [43]. Повышенная инфильтрация этих клеток может влиять на жизнеспособность гранулезоцитов, приводя к их апоптозу и атрезии фолликулов [44]. D. Liu и соавт. проанализировали транскриптом клеток гранулезы у женщин с ПНЯ и здоровых участниц для выявления потенциальных биомаркеров. Было обнаружено 10 дополнительных значимых для патогенеза новых генов: UBE2C, PBK, BUB1, CDC20, NUSAP1, CENPA, CCNB2, TOP2A, AURKB и FOXM1. Снижение экспрессии UBE2C в клетках гранулезы приводило к атрезии фолликулов: при его нокдауне происходило ингибирование пролиферации гранулезоцитов и их апоптоз с усилением активности маркеров программированной клеточной гибели. Следовательно, сниженная экспрессия UBE2C может инициировать дисфункцию гранулезы и, в последствии, развитие ПНЯ. Кроме того, в данной работе авторами было обнаружено, что продукт экспрессии UBE2C — убиквитинконъюгирующий фермент Е2С (UBE2C) был снижен и отрицательно коррелировал с моноцитами и макрофагами М1 [43]. Необходимы дальнейшие работы по изучению взаимосвязи между белком, кодируемым геном UBE2C и иммунной инфильтрацией при ПНЯ для определения нового возможного диагностического и прогностического маркера.

Подмножества лимфоцитов периферической крови являются важными параметрами для мониторинга иммунного ответа. На сегодняшний день большинство исследований субпопуляций этих клеток у пациенток с ранним истощением овариального резерва были сосредоточены на количестве каждой группы, однако их роль, функция и биологическое значение изучены недостаточно. C. Zhang и соавт. провели первый эксперимент, в котором использовался метод секвенирования одноклеточной РНК для изучения характеристик различных подмножеств лимфоцитов периферической крови у пациенток с ПНЯ [45]. Результаты показали, что состав нескольких клеточных субпопуляций, в основном моноцитов, NK-клеток и В-клеток, значительно отличался у женщин со сниженным овариальным резервом по сравнению со здоровыми участницами. Процент классических моноцитов и естественных киллеров оказался низким, а количество плазматических клеток было увеличено в основной группе. И хотя у пациенток с ПНЯ наблюдалось уменьшение количества NK-клеток, гены, связанные с их активностью (KLRC1, CD3E), были сверхэкспрессированы. Естественные киллеры являются важнейшим компонентом поддержания иммунитета. Но их роль остается недостаточно изученной в репродуктивных процессах.

В ряде работ обнаружен существенный вклад TNFα-зависимого сигнального пути в патогенез ПНЯ. TNF-α в основном продуцируется моноцитами, макрофагами, ДК, активированными В-лимфоцитами и Т-лимфоцитами. Он может связываться с двумя рецепторами (TNFR1 и TNFR2) и оказывать плейотропное действие на иммунитет и воспаление [46]. TNF-α играет ключевую роль в нескольких сигнальных путях, включая NF-κB, JAK/STAT, Akt, p38/MAPK, ERK и Wnt/β-катенин [47]. Многие исследователи рассматривают TNF-α и IL-1β при ПНЯ как показатели воспаления. IFN‐γ и TNF‐α напрямую приводят к дисфункции гранулезных клеток и атрезии фолликулов, а также развитию овариальной недостаточности [48]. В совокупности эти данные позволяют предположить, что аномалии иммунных клеток периферической крови могут быть вовлечены в этиологию заболевания, однако необходимо проведение исследований с большим объемом выборки.

Нарушения клеточного иммунитета являются неотъемлемой частью патогенеза ПНЯ. Т-лимфоциты — это самые важные эффекторы в иммунной системе. Следует отметить, что из-за существования различных подмножеств Т-клетки могут не только служить индукторами, но и ингибиторами аутоиммунных заболеваний. Известно об изменениях пропорции подгрупп Т-лимфоцитов, и весьма вероятно, что они связаны с возникновением и развитием ПНЯ [49]. Хотя этому вопросу уделялось огромное внимание, выводы о модификациях субпопуляций все еще противоречивы.

В многочисленных исследованиях было показано, что у пациенток с ПНЯ доля CD4+ Т-лимфоцитов (CD4+ клетки), поддерживающих воспаление, в периферической крови была повышена. Это позволяет предположить, что провоспалительный статус может быть фактором риска заболевания. Теоретически Treg-клетки могут подавлять потенциальную аутореактивную способность CD4+ клеток для поддержания баланса между провоспалительными и противовоспалительными свойствами. Однако в одной из работ было зарегистрировано снижение количества регуляторных Т-лимфоцитов и увеличение количества активированных CD4+ клеток при ПНЯ, что указывает на нарушение регуляции аутоиммунных реакций [50]. Основным объяснением может быть нестабильность Treg-клеток, способствующая развитию этого заболевания. Кроме того, мутация гена FOXP3, еще одного важного клеточного регулятора, может привести к их дисфункции, что в дальнейшем вызовет многочисленные аутоиммунные нарушения. В соответствии с функциональной недостаточностью Treg-клеток, уровни мРНК транскрипционного фактора белка FOXP3 в периферической крови больных ПНЯ были значительно снижены [51]. Проспективное контролируемое исследование показало, что у пациенток с идиопатической формой заболевания не наблюдалось изменений уровня CD4+ клеток. Однако процент регуляторных Т-лимфоцитов был значительно снижен, что было интерпретировано как механизм, потенцирующий аутоиммунный ответ [52].

Стоит отметить исследование Y. Wang и соавт., в котором изучалась возможность усиления функции Treg-клеток с помощью генной инженерии для повышения их эффективности при лечении ПНЯ [53]. В этой работе оценивали значение Pellino-1 — Е3-убиквитинированной лигазы (Peli1) в пролиферации и иммуносупрессивной функции регуляторных Т-лимфоцитов и терапевтический эффект этих клеток, сверхэкспрессирующих Peli1 при аутоиммунной ПНЯ. Вышеописанный белок отвечает за пролиферацию и активацию Т-лимфоцитов и тесно связан с некоторыми аутоиммунными заболеваниями, такими как рассеянный склероз, системная красная волчанка и ревматоидный артрит [54]. Было показано, что Peli1 слабо экспрессируется у пациенток с изучаемым заболеванием и, возможно, участвует в его патогенезе [55]. Результаты эксперимента под руководством Y. Wang выявили, что сверхэкспрессия данного белка способствовала клеточной пролиферации и усиливала иммуносупрессивную функцию Treg-клеток in vitro. После адаптивного переноса регуляторных Т-лимфоцитов, сверхэкспрессирующих Peli1, экспериментальным моделям (мыши) с аутоиммунной формой ПНЯ, скорость апоптоза гранулезных клеток яичников снизилась. Уровни ингибиторов воспаления: IL-10 и трансформирующего фактора роста-β, а также эстрадиола повысились. Количество примордиальных, первичных, вторичных и зрелых фолликулов восстановилось в определенной степени по сравнению с таковыми в контрольной группе [52]. Эти результаты могут дать новое представление о лечении ПНЯ и открыть перспективные терапевтические возможности.

В реализации широкого спектра процессов, протекающих в организме, цитокины принимают непосредственное участие. Данные биологически активные гормоноподобные белки определяют тип и длительность иммунного ответа, стимуляцию или подавление роста клеток, их дифференцировку, функциональную активность [56].

Тh1-клетки и макрофаги М1 продуцируют различные цитокины, включая IL-1α, IL-2, IL-6, IL-8, TNF-α и IFN-γ. Более того, Th17-клетки могут продуцировать IL-17 и IL-21. Эти цитокины являются важными маркерами воспалительных процессов. H. Yang и соавт. выявили повышенные уровни IL-1α в образцах сыворотки и фолликулярной жидкости у пациенток с ПНЯ по сравнению со здоровыми женщинами. Одновременно наблюдалось значительное усиление экспрессии апоптотических мРНК белка Bax, TNF-α и подавление экспрессии мРНК антиапоптотического пептида Bcl-2 [57]. На экспериментальных моделях и у пациенток с истощением овариального резерва исследователи обнаружили, что сывороточные концентрации провоспалительных цитокинов IL-6, IL-8, TNF-α, IL-17 и IFN-γ повышены, но снижена концентрация противовоспалительного цитокина IL-10, продуцируемого Treg-клетками, функция которого заключается в ингибировании активности Th1-клеток. Эти данные еще раз подчеркивают наличие сдвига провоспалительного статуса у больных ПНЯ и особую роль цитокинов при описываемой патологии. В клинической практике возможно проводить мониторинг уровней цитокинов с целью более ранней диагностики заболевания, тем самым максимально улучшая качество жизни и репродуктивные исходы у женщин [58].

Группа исследователей под руководством W. Zhang изучала вероятную связь развития ПНЯ с дефицитом IL-22. Последний является членом семейства цитокина IL-10, вырабатывается активированными Т-клетками, включая Th17 и Th22-клетки, а также врожденными лимфоидными клетками типа 3 (ILC3) и NK-клетками. Уровни IL-22 и IL-22-продуцирующих CD4+Т-лимфоцитов в сыворотке крови были значительно снижены в группе пациенток с ПНЯ по сравнению с контролем. Также данные показатели достоверно коррелировали с маркерами овариального резерва: АМГ, ФСГ, количеством антральных фолликулов по данным ультразвукового исследования [59]. Таким образом, эта работа впервые продемонстрировала связь между Th22-опосредованным дефицитом IL-22 и недостаточностью яичников, что открывает новые возможности для экзогенного введения IL-22 в качестве потенциального экспериментального лечения при ПНЯ.

Большинство ученых, занимающихся проблемой раннего истощения овариального резерва, склонны считать, что аномальные уровни иммунологических клеток и цитокинов приводят к снижению количества фолликулов в яичниках. Однако патологические механизмы до сих пор требуют дальнейшего изучения.

## ЗАКЛЮЧЕНИЕ

Таким образом, вышеописанные исследования позволяют утверждать, что роль иммунных нарушений в патогенезе некоторых форм аменореи велика. Воспаление является неотъемлемым компонентом СПЯ, хотя обычно это упускается из виду в клинической практике. Для поддержания функции яичников важно, чтобы уровни маркеров воспаления были сбалансированы; доказано, что различные концентрации провоспалительных и противовоспалительных цитокинов способствуют дисфункции яичников и неправильному созреванию фолликулов. Не менее важно адекватное функционирование процессов клеточного иммунитета.

ПНЯ — гетерогенное и многофакторное заболевание, при котором идиопатическая форма составляет большинство случаев, а их этиология до сих пор неизвестна. Современные исследования показывают, что иммунная система играет ключевую роль в патогенезе данной нозологии. Яичники являются частой мишенью аутоиммунных атак. Накапливающиеся данные указывают на то, что, помимо индукции синтеза аутоантител, патология клеточного иммунитета занимает не последнее место при ПНЯ. Однако точные механизмы, лежащие в основе иммунопатогенеза снижения овариального резерва, приводящего к бесплодию и развитию множественных менопаузальных проявлений, не до конца исследованы.

На основании вышеизложенного материала можно сделать вывод о наличии единых звеньев нарушений в иммунной системе таких отличных друг от друга заболеваний, как СПЯ и ПНЯ.

Несмотря на многочисленные достижения в изучении иммунопатологии, применение лекарственных средств, направленных на это звено патогенеза, недостаточно изучено, что создает большой плацдарм для разработки и внедрения инновационных терапевтических средств.

## ДОПОЛНИТЕЛЬНАЯ ИНФОРМАЦИЯ

Источники финансирования. Работа выполнена в рамках государственного задания №123021300169-4 «Эпигенетические предикторы и метаболомная составляющая аменореи различного генеза у женщин репродуктивного возраста», 2023–2025 гг.

Конфликт интересов. Авторы декларируют отсутствие явных и потенциальных конфликтов интересов, связанных с содержанием настоящей статьи.

Участие авторов. Все авторы одобрили финальную версию статьи перед публикацией, выразили согласие нести ответственность за все аспекты работы, подразумевающую надлежащее изучение и решение вопросов, связанных с точностью или добросовестностью любой части работы.
